# Influence of Birth Weight on the Renal Development and Kidney Diseases in Adulthood: Experimental and Clinical Evidence

**DOI:** 10.1155/2012/608025

**Published:** 2012-06-17

**Authors:** Maria C. P. Franco, Vanessa Oliveira, Beatriz Ponzio, Marina Rangel, Zaira Palomino, Frida Zaladek Gil

**Affiliations:** ^1^Division of Nephrology, School of Medicine, Federal University of São Paulo, 04023-062 São Paulo, SP, Brazil; ^2^Department of Physiology, School of Medicine, Federal University of São Paulo, 04023-062 São Paulo, SP, Brazil; ^3^Department of Molecular Cardiology, Cleveland Clinic, Cleveland, OH 44195, USA

## Abstract

Several clinical and experimental studies support the hypothesis that foetal programming is an important determinant of nephropathy, hypertension, coronary heart disease, and type 2 diabetes during adulthood. In this paper, the renal repercussions of foetal programming are emphasised, and the physiopathological mechanisms are discussed. The programming of renal diseases is detailed based on the findings of kidney development and functional parameters.

## 1. Introduction

Foetal growth is both complex and delicate. This critical phase of development is determined not only by substrate availability but also by the integrity of the physiologic processes necessary to ensure the transfer of nutrients and oxygen to the foetus. Impairment of the intrauterine growth environment during critical periods may result in perturbations of development, characterised by intrauterine restricted growth (IUGR) and low birth weight (LBW). In fact, Professor David Barker and colleagues in Southampton, United Kingdom, originally proposed the hypothesis that several chronic adult diseases can be programmed in early life [1]. This programming concept is based on the idea that some stimulus or injury during these critical phases of development can result in permanent physiologic and metabolic changes and produce persistent effects throughout life [1, 2]. Further, Hales and Barker suggested the *Thrifty Phenotype Hypothesis* to explain the biological basis of the foetal origin hypotheses. In this context, impairment of foetal environment can result in a physiologically adaptive response that promotes early survival at the detriment of later health [3]. These foetal adaptive responses to a suboptimal intrauterine environment optimise the growth of key body organs to the detriment of others and lead to an altered postnatal metabolism. The main basis of this hypothesis is that the deleterious effects are more pronounced when there is a significant difference between early nutritional deficiency and later nutritional intake [3, 4]. During the last few decades, several clinical and experimental studies have confirmed and extended these hypotheses, suggesting that LBW is significantly correlated to the development of hypertension, cardiovascular disease, and type 2 diabetes [1–6]. 

## 2. Foetal Programming of Renal Development and Kidney Disease

In humans, nephrogenesis is completed at approximately 32nd–34th week of gestation, and therefore, a nephron deficit present at birth would persist throughout life. A kidney with a congenitally reduced nephron number has less functional reserve and becomes more susceptible to subsequent renal injury and functional decline. There is evidence that individuals with history of LBW have a congenital deficit of total nephron amount [7–11]. These observations indicated that during the renal developmental period, the kidney can be influenced quite dramatically by alterations in the intrauterine environment that lead to impairment of nephrogenesis. In fact, an ultrasound study showed that in LBW individuals appeared to have thinner kidneys of normal length, suggesting decreased nephron number [8]. Other studies have shown a direct relation between birth weight and the number of nephrons, with 250,000 more glomeruli per kidney per kilogram increase in birth weight [9, 12]. In addition to these findings, early catch-up kidney growth was observed in small-for-gestational-age (SGA) infants, suggesting either an accelerated renal maturation process or early compensatory kidney hypertrophy in these infants [13]. Moreover, Orskov et al. [14] found that LBW contributes to phenotypic variability in the progression of renal disease in patients with autosomal dominant polycystic kidney disease. Another study showed that children with congenital chronic kidney disease (CKD) had a higher rate of prematurity and LBW than newborns with hereditary or acquired CKD [15]. Similar results have been reported by other authors who observed increased susceptibility to diabetic nephropathy and more rapid progression of nephrotic syndrome, chronic pyelonephritis, and IgA nephropathy among LBW subjects [11, 16–18]. There is also increasing evidence available concerning the effect of IUGR on the impairment of renal function in children and adults [19–24]. The assessment of renal function is important to detect the extension and progression of nephropathy. Previous studies in different populations, such as Australian Aboriginals and Pima Indians, have reported that the prevalence of albuminuria was higher in individuals who had LBW [16, 20, 21]. Furthermore, a recent study demonstrated that microalbuminuria levels were significantly higher in IUGR compared with normal infants at a mean age of 18 months [22]. It has been reported that renal clearance of amikacin on the first day of life is lower in LBW neonates, suggesting that IUGR impairs GFR on the first day of life [23]. Moreover, evidence highlighting the impact of LBW on renal function was described in SGA children with high levels of cystatin C [24]. In this context, the LBW appears to be associated with early impairment of renal function and this association is consistent with the high incidence of lower numbers of nephrons in cases of foetal growth restriction.

The hypothesis that IUGR programmed to inappropriate renal development has been corroborated by experimental studies. Several data derived from animal studies indicate that changes in foetal environment may affect renal development. This hypothesis receives support from observations of both global nutrient and protein restriction during pregnancy, resulting in reduced nephron number and impaired renal function in adult offspring [25–28]. In fact, Merlet-Benichou et al. [25] described a reduction in nephron number in offspring submitted to substantial protein restriction in utero. Another study showed that a 50% protein restriction during pregnancy produces offspring with a reduced number of glomeruli, glomerular enlargement, and hypertension in adulthood [27]. In addition, Langley-Evans et al. [26] demonstrated in rats that prenatal exposure to a maternal diet low in protein in mid-to-late gestation induces impaired nephrogenesis and hypertension in adult life. Nwagwu et al. [28] showed that a low-protein diet in utero induced increased blood urea, urinary output, and urinary albumin excretion in the resulting offspring. It has also been shown that the induction of IUGR by uteroplacental insufficiency produces a significant reduction in glomerular number in full-term foetal kidneys [4]. It has been suggested that some aspects of nephrogenesis, which begins around day 12 of gestation and is not complete until 8 days after birth, are maladaptive processes leading to the subsequent development of hypertension. Additional evidence comes from some studies developed by Lucas et al. [29–31]. These authors demonstrated that when pregnant rats were subjected to 50% food restriction during the first, second half, or throughout their entire pregnancy, the renal function of their offspring was impaired 3 months after birth. Morphometric evaluation showed that the number of glomeruli was significantly decreased in both newborn and adult offspring, irrespective of the period in which the restriction was imposed. Glomerular diameter showed a significant increase in every studied group, which characterised a compensatory hypertrophy in the remaining nephrons. These findings led us to hypothesise that intrauterine malnutrition could be a determinant for the early appearance of glomerulosclerosis in adult life. In a subsequent study, when renal function studies were performed in 18-month-old rats submitted to intrauterine malnutrition, a significant decrease in GFR and renal plasma flow (RPF) levels was observed. Moreover, histological evaluation of kidney sections showed a marked increase in glomerulosclerosis and tubulointerstitial lesions in these rats. Immunohistochemical studies revealed that an accelerated process of glomerulosclerosis took place in these rats, with high expression levels of fibronectin, desmin, and L-actin in the glomeruli, Bowman's capsules, and interstitial areas. These findings led us to conclude that age-induced renal changes could be accelerated in this IUGR model, with renal structural changes occurring early in life. The data reported by Mesquita et al. [32] corroborate this hypothesis. These authors found enlargement of podocytes in IUGR offspring, indicating that these morphological changes could be attributed to an adaptation to the reduced nephron number and, consequently, to glomerular hyperfiltration and overflow in these animals. Recently, Luyckx et al. [33] showed that acceleration of renal senescence is higher in LBW rats with subsequent rapid catch-up growth. These observations indicated that during the foetal developmental, the kidney can be influenced quite dramatically by deleterious alterations in the intrauterine environment. This can then lead to reduction in nephron endowment and, later, renal diseases.

## 3. Possible Pathways Leading to Foetal Programming of the Kidney Diseases: The Role of the Intrarenal Renin-Angiotensin System, Renal Sodium Transport, and Apoptosis

It is well established that the intrarenal renin-angiotensin system (RAS) plays an important role in the normal morphological development of the kidney [34]. In fact, all components of the RAS are expressed early in gestation in the rat and human meso- and metanephrons [4, 10, 20, 34]. There is considerable evidence that intrarenal RAS is affected by an unfavourable foetal environment. Some studies demonstrated that renal renin and angiotensin II (Ang II) mRNA levels were significantly reduced in newborns submitted to a low-protein diets *inutero,* suggesting that maternal malnutrition promoted the suppression of newborn intrarenal RAS, and that may affect nephrogenesis, resulting in a lower nephron number [4, 35, 36]. Another study found in early foetal life that AT_1_ receptor expression was increased, whereas AT_2_ declined in kidneys isolated from IUGR rats [37]. In adult IUGR offspring, elevated mRNA levels of intrarenal angiotensin converting enzyme (ACE), renin, and angiotensinogen were found, but no changes in intrarenal Ang II levels were documented [38]. Studies have demonstrated that inadequate protein during pregnancy promoted reduced nephron number and increased AT_1_ and AT_2_ protein levels in the cortex, but no significant change in the renal Ang I and Ang II levels in the early postnatal life of offspring [39–41]. Recently, Mesquita et al. [42] reported a total absence of AT_2_ in the glomeruli, and this receptor was preferentially associated with the intercalated cells of the distal and collecting segments of adult offspring submitted to a low-protein diet *in utero*. Moreover, the same authors also found that the AT_1_ and AT_2_ receptors were downregulated in association with alterations in the JAK-2/SOCS3 pathways in these animals. Chou et al. [43] demonstrated that overactivity of chymase may result in increased intrarenal Ang II concentration in the IUGR kidney. Despite the discrepancies regarding intrarenal RAS among the cited groups, which may be related to differences in the feeding protocol used, severity and duration of nutritional deficiencies and changes in the RAS programmed during foetal life may be responsible for the renal alterations observed in these animals.

Experimental studies support the hypothesis that foetal programming is also correlated with regulation of sodium transporters [44, 45]. In fact, Alwasel and Ashton [46] described an absence of the Na^+^/K^+^-ATPase-*α*1 catalytic subunit in the kidneys of IUGR offspring during early postnatal life, while other authors showed an upregulation of protein and mRNA expression of the *α*1 and *β*1 subunits of the Na^+^/K^+^-ATPase during adulthood [32, 47]. These alterations could result in a high capacity to retain salt and water and expansion of the intravascular compartment. It is known that Ang II is a powerful sodium-retaining hormone, which acts directly on renal tubular transport. Indeed, evidence revealed that Ang II, via AT2 receptors, is a potent inhibitor of Na^+^/K^+^-ATPase [40], and it is possible that the downregulation of this receptor observed in IUGR offspring might lead to a lack of inhibition of Na^+^/K^+^-ATPase, explaining their low sodium excretion rate. Moreover, Manning et al. [48] demonstrated that renal bumetanide-sensitive cotransporter (NKCC2), thiazide-sensitive cotransporter (NCC) mRNA, and protein levels were increased in the thick ascending limb and distal convoluted tubule of adult IUGR offspring. However, these same authors also noted that protein concentrations of the Na^+^/H^+^ exchanger 3 (NHE3) and all of the ENaC (epithelial sodium channel) subunit proteins were unchanged in these offspring, suggesting that proximal tubule sodium transport and the fraction of sodium excretion mediated by this exchanger are not affected by foetal programming [48]. Experimental models provide a broad overview of renal sodium transport in IUGR offspring and indicate upregulation of two sodium transporters in specific segments of the nephron. Therefore, when the kidney is programmed, the nephrons have inappropriate renal sodium transporter regulation, and this contributes to alterations in sodium handling. These alterations lead to a lower rate of urinary sodium excretion and sodium retention. Whether there is another nephron segment or other sodium transporters affected by foetal programming remains unclear; however, this topic provides a promising field for future investigation.

Some studies have shown the importance of the perfect balance between cell proliferation and apoptosis during kidney development and its potential role on foetal programming [49–51]. The kidney development is an intricate process named “branching morphogenesis,” which involves several signaling molecules and transcription and growth factors; any dysregulation in this crucial process can lead to a change in cell proliferation, apoptosis, and impaired nephrogenesis. Clinical and experimental studies have pointed a significant association between reduced kidney size at birth and genetic polymorphisms of the paired-box gene 2 (PAX2) and protein tyrosine kinase receptor (RET) [52, 53]. The presence of common polymorphic variants of these genes involved in renal branching morphogenesis, can confer high susceptibility to develop renal disease in LBW individuals.

Experimental models of IUGR were associated with increased apoptosis of the glomerular cells, interstitial cells, and tubule epithelial cells [36]. This process could be due to the downregulation of antiapoptotic factors, such as PAX-2 or Bcl-2, and/or the upregulation of proapoptotic factors, including Bax and p53 [50, 51]. Studies carried out by Welham et al. [50, 51] showed that IUGR rats had a reduction in nephron number and elevated deletion of precursors at the start of metanephric development. These same authors also described high expression of both Bax and Bcl-2 in IUGR rats, and this increase was greater in the proapoptotic gene (Bax) than the antiapoptotic gene (Bcl-2), which leads to increased death of metanephric precursor cells. Moreover, bilateral partial ligation of the uterine arteries during pregnancy resulted in p53 hypomethylation and elevated expression of Bax and p53 as well as a reduction in Bcl-2 mRNA [54]. The increased ratio between proapoptotic and antiapoptotic factors promotes renal cellular apoptosis in these offspring [36, 54]. Epigenetic pathways could be a mechanism whereby foetal programming alters the methylation status and transcriptional rate of some genes, including Bcl-2 and Bax, leading to foetal renal apoptosis and permanent loss of glomeruli [32, 55].

## 4. Conclusions

There is strong evidence that the kidney can be influenced by deleterious alterations during foetal life. Clinical and experimental data suggest that an inappropriate intrauterine environment may permanently modify the structure of the kidney. This modification is evidenced not only by reduced nephron number, but also by a compensatory maladaptive change that occurs intrarenally when nephrogenesis is compromised. It must be recognised that the high risk for kidney disease associated with foetal programming could be a direct consequence of impaired nephrogenesis or a cumulative process superimposed on type 2 diabetes and/or cardiovascular diseases. Future studies should be conducted to elucidate the molecular pathways involved in this phenomenon.
